# Elucidating the anti-aging mechanism of Si Jun Zi Tang by integrating network pharmacology and experimental validation *in vivo*

**DOI:** 10.18632/aging.204055

**Published:** 2022-05-10

**Authors:** Yang Yuan, Yanghuan Zhang, Runzi Zheng, Hongjun Yuan, Ruoyu Zhou, Shuting Jia, Jing Liu

**Affiliations:** 1 Laboratory of Molecular Genetics of Aging and Tumor, Medical School, Kunming University of Science and Technology, Kunming 650500, Yunnan, China

**Keywords:** SJZT, aging, network pharmacology, experimental validation

## Abstract

Si Jun Zi Tang (SJZT) is a classic Traditional Chinese Medicine (TCM) prescription used to treat aging-related diseases. However, the potential molecular mechanisms of the anti-aging effects of the bioactive compounds and their targets remain elusive. In this study, we combined network pharmacology and molecular docking with *in vivo* experiments to elucidate the anti-aging molecular mechanism of SJZT. A series of network pharmacology strategies were used to predict potential targets and therapeutic mechanisms of SJZT, including compound screening, pathway enrichment analysis and molecular docking studies. Based on the network pharmacology predictions and observation of outward signs of aging, the expression levels of selected genes and proteins and possible key targets were subsequently validated and analysed using qRT-PCR and immunoblotting. Using a data mining approach, 235 effective targets of SJZT and aging were obtained. AKT1, STAT3, JUN, MAPK3, TP53, MAPK1, TNF, RELA, MAPK14 and IL6 were identified as core genes in the Protein-Protein Interaction Networks (PPI) analysis. The results of the effective target Gene Ontology (Go) functional enrichment analysis suggested that SJZT may be involved aging and antiapoptotic biological processes. The Kyoto Encyclopedia of Genes and Genomes (KEGG) enrichment analysis indicated that the anti-aging mechanism of SJZT may be associated with the PI3K-AKT and P38 MAPK signalling pathways. Molecular docking analysis suggested that kaempferol and quercetin could fit in the binding pockets of the core targets. In addition, SJZT alleviated the aging symptoms of mice such as osteoporosis and hair loss. In conclusion, the anti-aging effect of SJZT was associated with the inhibition of the PI3K-AKT and P38 MAPK signalling pathways, and these findings were consistent with the network pharmacology prediction.

## INTRODUCTION

Aging is a biological process influenced by a variety of complex factors, such as heredity, environmental conditions and lifestyle [1]. With the increase of age, the physiological integrity of body organs gradually declines leads to organ fragility, and aging-related diseases such as loss of skeletal muscle mass, cardiac hypertrophy, heart function, decline and osteoporosis [2, 3]. Aging involves in many complex factors, single target or single drug cannot fundamentally delay the progression of aging and aging-related diseases. Traditional Chinese Medicines (TCMs) feature multiple components, targets and pathways and can be used to combat aging. Si Jun Zi Tang (SJZT) is composed of four herbs, including *Panax ginseng C. A. Mey, Atractylodes macrocephala* Koidz, *Poria cocos* (Schw.) Wolf and Glycyrrhizae [4]. The prescription has also been found to have anti-aging effects; for example, it can ameliorate spleen and liver aging in mice [5, 6]. Jin et al. also found that Si Jun Zi Tang alleviates airway inflammation in mice with chronic asthma through the mTORC1 pathway [7]. Liang et al. also preliminarily analysed the anti-aging mechanism of Si Jun Zi Tang by using network pharmacology [8], but only a single data source was used, experimental verification was lacking. However, the active ingredients of SJZT and its specific mechanism in the aging process have not been systematically elucidated. In fact, the mechanism of action of TCM compounds have always been unresolved because TCM formulations are complex systems with multiple components, multiple targets and multiple pathways [9].

Network pharmacology is a new research tool based on the interdisciplinary theory of pharmacology and biology and includes virtual prediction, protein interaction, molecular docking, topological network and *in silico* analyses [10]. The *in silico* technique is a technique that simulates biological experiments completely on a computer and has been widely used in target identification and potential drug design [11, 12]. Unlike the traditional pharmacology research strategy, network pharmacology does not study the interaction between a single disease, a single target, and a single drug in isolation but measures the complex “ingredient- target-disease” network relationship from a systematic and holistic perspective [13, 14]. Therefore, it has been applied to predict the effects of herbal compounds on age-related diseases. With this tool, He et al. successfully predicted the effective components, potential targets and possible pathways of Liu Wei Di Huang pills in the treatment of type 2 diabetes [15]. Liu et al. successfully predicted the anti-aging mechanism of resveratrol [16]. However, these studies lacked validation of key targets and pathways at the cellular or animal experimental level.

In this study, we used a network pharmacology method to analyse the interactions among active compounds, potential targets and target diseases of SJZT. According to the preliminary analysis results, we studied the mechanism by which SJZT regulates the P38MAPK and PI3K-AKT signalling pathways in the treatment of aging in a mouse model of natural aging. And these findings were consistent with the network pharmacology prediction.

## MATERIALS AND METHODS

### SJZT active compound screening

The chemical compounds of the four herbs of SJZT were acquired from the TCMSP database (http://tcmspw.com/tcmsp.php) [17]. TCMSP provides important data on the absorption, distribution, metabolism and excretion (ADME) characteristics of TCMs, including oral bioavailability (OB) and druglikeness (DL) data [18]. A chemical compound is considered active if it meets the following criteria: OB ≥ 30%, Caco-2 permeability ≥ −0.4, and DL ≥ 0.18 [19–21]. In this study, we regarded compounds with OB≥30%, DL≥0.18 and Caco-2 permeability ≥ −0.4 as active candidate compounds.

### Potential targets identification

Identification of putative targets of SZJT candidate compounds was performed with TCMSP and Drugbank (https://www.drugbank.ca/) [20, 22]. The target protein names were transformed into the corresponding gene symbols by the UniProt database (https://www.uniprot.org) [23]. “Aging” was used as a key word to search for targets related to aging. The predicted targets of aging were collected from the GeneCards database (https://www.genecards.org, verb4.9.0) [24], NCBI (https://www.ncbi.nlm.nih.gov/gene) [25] and OMIM databases (http://www.omim.org/, updated December, 30, 2020) [26]. The species was set to be “Homo *Sapiens*”. Then, aging related genes found in the three databases were merged, the replicate genes were deleted.

### Construction of the targeted aging network of SJZT

The overlapping genes associated with both SJZT active compounds and aging were considered as hub genes. Then the SJZT-target-aging network was constructed using Cytoscape 3.7.2 software. The hub genes were put into the STRING (https://string-db.org/, V11.0) database [27], with the species limited to “*Homo sapiens*” and a setting of a combined score>0.900, to construct the protein–protein interaction (PPI) network. The results were downloaded as TSV format file from the STRING database and imported into the Cytoscape V3.7.2 for analysis [23]. CytoHubba, a plugin of Cytoscape V3.7.2, was used to calculate the degree centrality [28]. Larger protein targets with more linked nodes had higher degrees, participated in more biological functions [29, 30], and were more likely to be therapeutic targets. The top ten core targets were screened to determine the accuracy using the critical targets of the molecular docking method.

### Bioinformatics analysis based on target genes

GO and KEGG pathway analyses of the SJZT hub targets were performed using the DAVID 6.8 database (https://david.ncifcrf.gov/tools.jsp). The species was set to be “*Homo Sapiens*”. The GO functional analysis was performed for three categories, including the biological process (BP), molecular function (MF), and cellular component (CC) categories [31]. The GO bar chart results were drawn by Graph Pad Prism V8.0.1. The KEGG pathway enrichment results were visualized as a bubble chart with online bioinformatics tools (http://www.bioinformatics.com.cn/). In addition, a false discovery rate (FDR) <0.05 was considered to indicate significance.

### Molecular docking analysis

Molecular docking was performed with AutoDockTools V1.5.6 software to validate compound-target interactions [32]. First, we prepared receptor proteins. The crystal structures of core proteins were obtained from the Protein Data Bank (PDB) database (http://www.rcsb.org/). PyMol software was used to optimize the structures of receptor proteins by removing water molecules. Subsequently, hydrogens were added to the proteins and the charges were calculated with AutoDockTools V1.5.6 software and exported in PDBQT format.

Next, we prepared the ligand small molecule compounds. The SDF files of the core compound structures were downloaded from the PubChem database (https://pubchem.ncbi.nlm.nih.gov/). Then, we used Chem3D V15.1 software to optimize the structures and saved them in MOL2 format. Subsequently, the MOL2 format files of the bioactive compounds were imported into the AutoDockTools V1.5.6 software to adjust the charges, determine the roots of the ligands, and select the ligands’ rotatable bonds, and the results were saved in PDBQT format.

AutoDockTools was used to construct the binding pockets for molecular docking. We searched for the active site information of receptor proteins in the reported experimental results in the literature. Then center coordinates of the active pocket were adjusted according to the protein shape. And, the relevant coordinates of the docking box were determined. Finally, AutoDock Vina was used to semi-flexibly dock the core compounds to the core target proteins. The docking model with the lowest binding free energy was selected to analyse the final conformation. OpenBabel software was used to convert the PDBQT format to PDB format. PyMOL software was used to visualize the binding conformation of the ligand and receptor. Then, the best conformation was saved. The PDB format was entered into the Protein–Ligand Interaction Profiler (PLIP) website (https://plip-tool.biotec.tu-dresden.de/plip-web/plip/index). The website was used to visualize the docking mode of each compound-target interaction [33].

### Drug administration in the animal model

The individual herbs were provided by Jiang Yin Tian Jiang Pharmaceutical Company. The animal experiments were approved by the Experimental Animal Ethics Committee of the KUST Medical school and conformed to the principles of animal protection, animal welfare and ethics and the relevant national guidelines. The mice were raised in standard conditions at 23° C ± 2° C and 55% ± 15% humidity on a 12 h light dark cycle, and fed a normal diet. The 16-month-old mice were randomly divided into 2 groups and oral administration (10g/kg/day) with SJZT or saline. Two-month-old mice were used as the young control group. The body weights of all groups were recorded every three days. After 4 weeks, the mice were sacrificed to collect tissue for further analysis.

### Micro-CT analysis

Isolated shin bones were placed in a 48-mm specimen holder and then subjected to μCT scanning (Model LaTheta LCT-200, Hitachi-Aloka, Tokyo, Japan). Briefly, an overview scan of the whole isolated bone was first performed [34]. Then, the opposite ends of isolated bone were scanned to quantify cortical and spongy bones. The total bone thickness was calculated automatically by the Latheta software (version 3.56). 3D reconstruction was performed with VGSTUDIO MAX 3.0.

### Immunoblotting

Liver tissues were lysed with RIPA buffer containing 1 mmol/L phenylmethanesulfonyl fluoride (PMSF). The protein concentration was quantified with a BCA protein assay kit. Then, 20 μg of protein was separated by SDS–PAGE electrophoresis, and then proteins were transferred to a methanol-preactivated PVDF membrane. After blocking at room temperature for 2 h using 2% bovine serum albumin with gentle shaking, the membranes were incubated with primary antibodies (1:1000) overnight at 4° C. The membranes were washed and incubated with secondary antibodies for 2 h at room temperature. The PVDF membranes were visualized with an enhanced chemiluminescence (ECL) detection system (Tanon 5200, China). The primary antibodies (anti-STAT3, anti-P-STAT3, anti-NF-κB p65, anti-p-anti-NF-κB p65, anti-P38 MAPK, anti-p-P38 MAPK, anti-AKT and anti-p-AKT) were purchased from Cell Signaling Technology. The primary antibodies anti-P21 and anti-Bcl2) were purchased from BD. The primary antibodies anti-α-Tubulin and anti-β-actin were purchased from Santa Cruz, and the primary antibody anti-P53 was purchased from Abcam.

### Real-time quantitative polymerase chain reaction

To detect mRNA expression, total RNA was extracted from mouse liver tissue using 1 mL of TRIzol reagent (Invitrogen). The total RNA concentration was determined by spectrophotometry at 260 nm, and RNA integrity was assessed by agarose gel electrophoresis by the 28S and 18S RNA bands. One microgram of total RNA was reverse transcribed into cDNA using PrimeScript RT Master Mix (Perfect Real Time, TaKaRa, Japan), which was used in subsequent real-time qPCRs. Real-time qPCR was performed using SYBR Green (Roche) with a 7300 Real-Time PCR System (Applied Biosystems). The expression levels of mRNAs of interest were normalized to GAPDH expression levels. The PCR primers were as follows: *P16*, 5’-TAGTCCTTCCTACCCCAATTTCC-3’-(forward), and 5’-TTGGTCCTTAGCCACTCCTTC-3’-(reverse). *IL-6*, 5’-TACCCCGATTCAGGTGAT-3’-(forward), 5’-TTGAGCAGAAGAGCTGCTACGT-3’-(reverse). And *GAPDH*, 5’-AGGTCGGTGTGAACGGATTTG-3’-(forward), and GAPDH -5’-TGTAGACCATGTAGTTGAGGTCA-3’-(reverse). We used the 2^-ΔΔCt method to calculate the expression of mRNA. All reactions were repeated 3 times.

### Statistical analysis:

All data are expressed as the mean ± standard deviation (SD) and were analysed by one-way analysis of variance (ANOVA) followed by Dunnett’s multiple comparison test. Statistical analysis was performed using GraphPad Prism software. Statistical significance was considered significant at ^*^p <0.05, ^**^p <0.01 and ^***^p <0.001.

## RESULTS

### Screening of active compounds and potential targets

**Figure 1:**
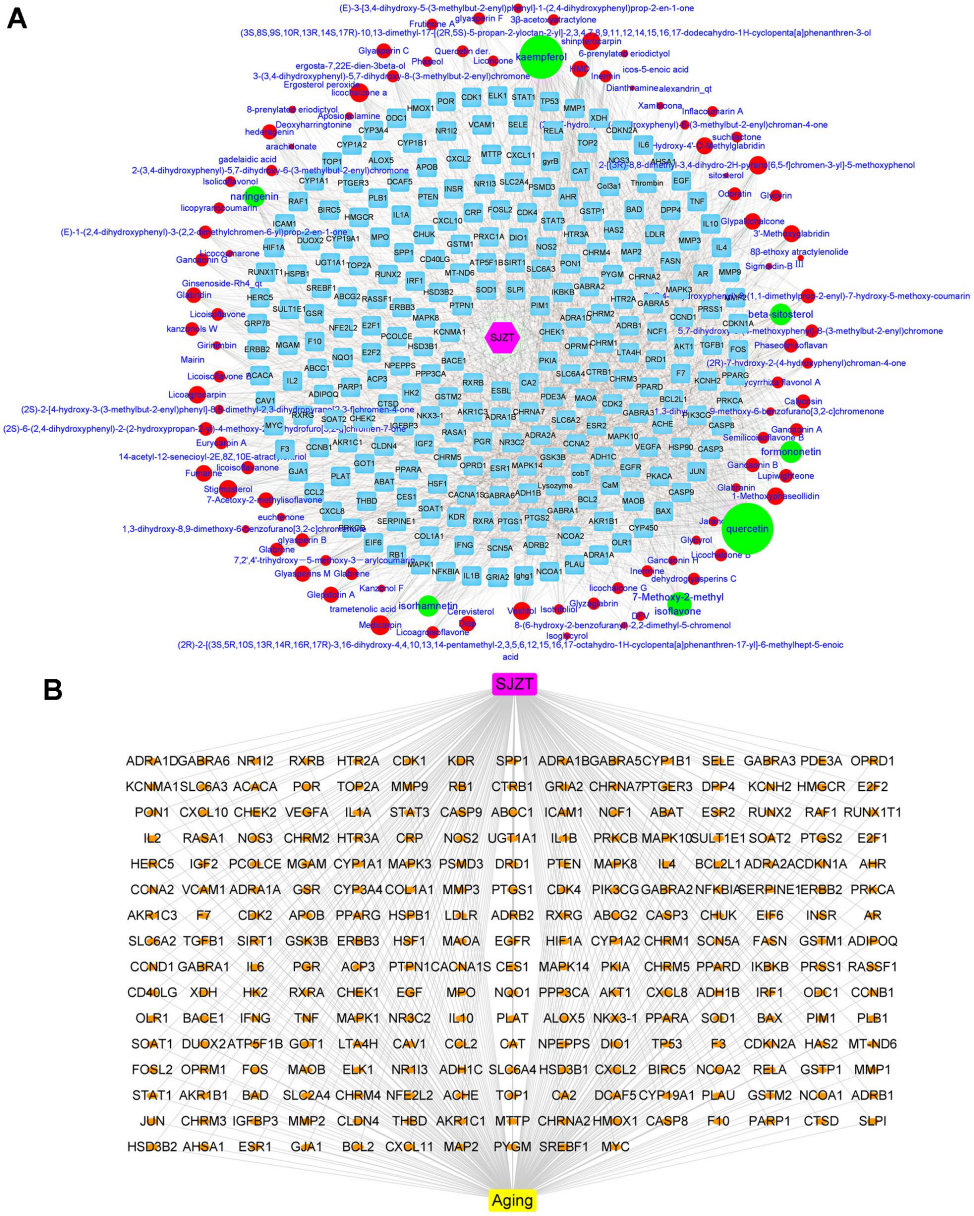
**Determination of active components of SJZT and common targets related to aging.** (**A**) SJZT-compounds-target network: The blue round rectangle stand for targets of SJZT compounds, red ellipse and green ellipse stand for SJZT compounds, the edge represented the interaction between nodes, degree indicated nodes sizes stand for interacting with the number of targets. Pink Hexagonal represents SJZT formula. (**B**) SJZT-target-aging network. The SJZT-target-aging network contains 241 nodes and 478 edges.

The bioactive components of the four herbs in SJZT were collected through the TCMSP database. There were 131 bioactive compounds that met the ADME parameters, which were OB≥30%, DL≥0.18 and Caco-2 permeability ≥ −0.4 (Supplementary Table 1). After removing duplicate targets, 249 targets of SJZT bioactive components were obtained from the TCMSP and converted into symbols of corresponding genes by using the UniProt database (Supplementary Table 2). To obtain a better understanding of the complex relationships between SJZT compounds and their targets, an SJZT-compound-target network was constructed. As shown in Figure 1A, the network included 359 nodes and 2040 edges. In this network, we found some unique compounds with higher degrees compounds, such as quercetin and kaempferol. Therefore, we identified that kaempferol and quercetin play key roles in the effects of SZJT. After the SJZT targets were compared with the 24553 aging-related candidate target database, 235 target genes were selected as hub genes and for the subsequent research. (Figure 1B).

### PPI network and screening of key targets

**Figure 2:**
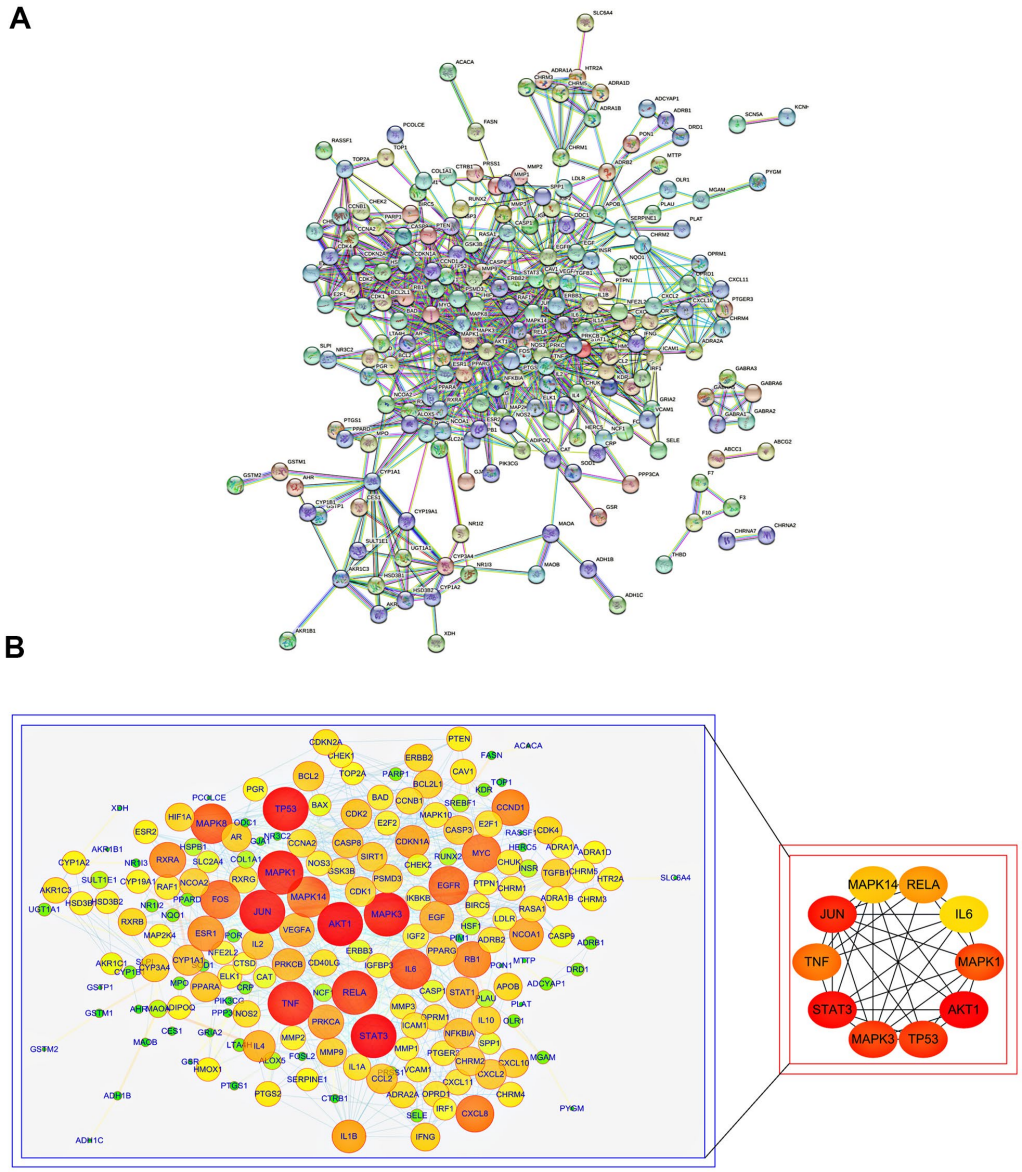
**PPI analysis of common targets of SJZT to identify core targets.** (**A**) PPI network of SJZT anti-aging related protein from STRING database. (**B**) Higher degrees indicated larger node sizes and the edge thickness represents the connection score. Screening of the Top 10 core targets. Nodes size and red color depth are proportional to their degree.

To further understand the molecular mechanism of aging and the functions of specific proteins, the STRING database was used to construct an SJZT-aging PPI network (Figure 2A). According to the topology analysis, the targets (degree score≥106), in order of degree score from high to low, were as follows: AKT1, STAT3, JUN, MAPK3, TP53, MAPK1, TNF, RELA/p65, MAPK14 and IL6. These ten target genes interact most closely with other targets. The results suggest that AKT1, STAT3, JUN, MAPK3, TP53, MAPK1, TNF, RELA/p65, MAPK14 and IL6 may be the core targets of the hub genes (Figure 2B). In the follow-up, it was necessary to further determine the core targets according to with results of molecular docking. The results provide a basis for determining the anti-aging effects of kaempferol and quercetin and the reliability of the virtual prediction of the molecular docking method.

### Molecular docking analysis

**Figure 3:**
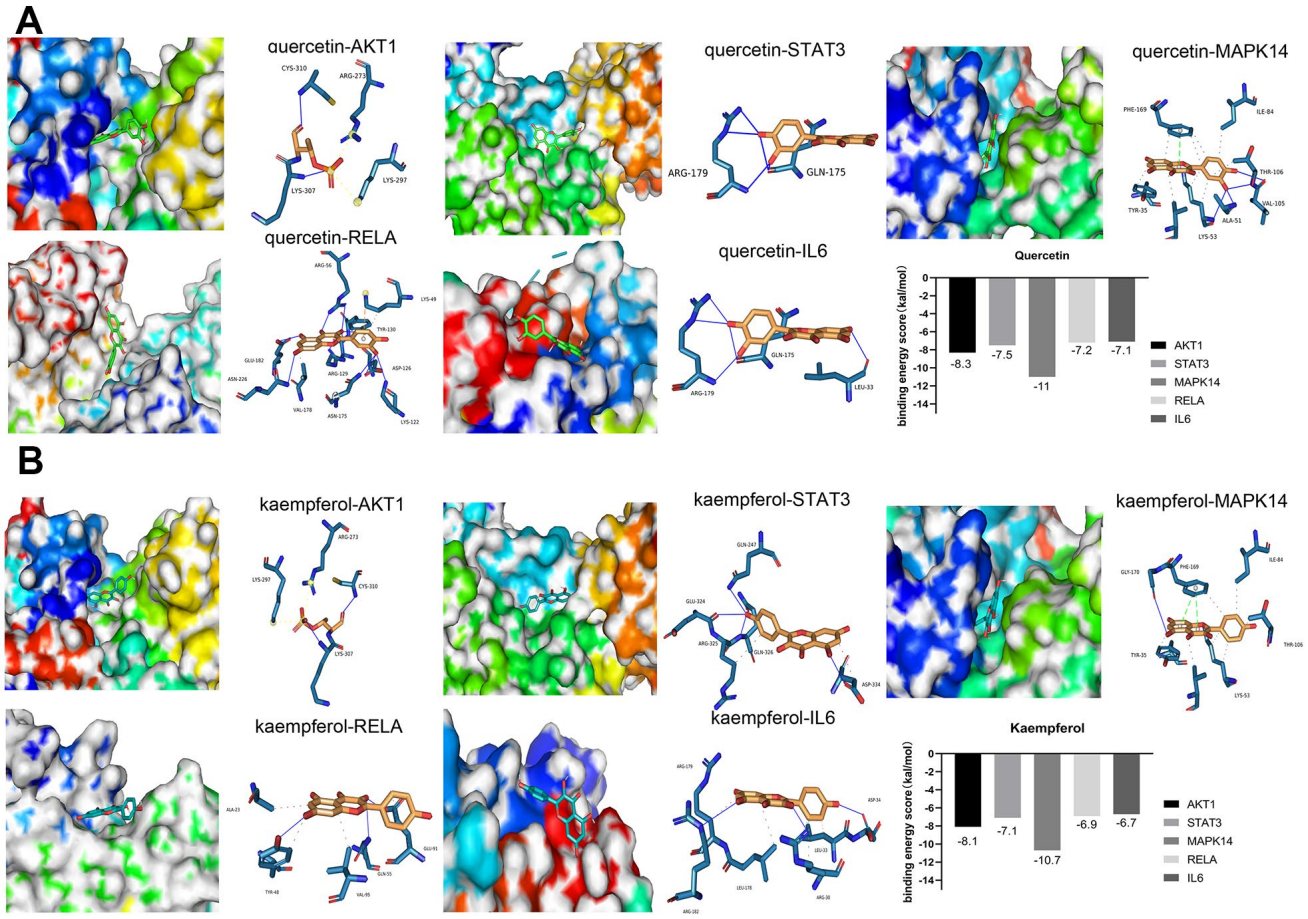
**Molecular docking between small molecule ligands and core targets protein.** The 3D surface structure of protein receptors and small molecular ligands is shown on the left. The right side shows the binding pattern of the small molecular ligand to the core target protein. AKT1-PDBID: 4gv1, STAT3-PDBID: 6njs, MAPK14-PDBID: 3Zs5, RELA-PDBID: 6nv2, IL6-PDBID: 1alu. (**A**) Quercetin (**B**) Kaempferol.

To improve the accuracy of the connection between small molecular compounds and the core target proteins, we used molecular docking to evaluate the interactions between compounds and target proteins. It is generally believed that when the binding energy of a ligand and receptor is <−4 kcal/mol, there is potential binding activity between the ligand and receptor [35]. A binding energy score less than −5 kcal/mol suggests strong binding between the ligand and receptor [36, 37]. The results of molecular docking showed that kaempferol and quercetin, two important compounds in SJZT, had good affinity for the top ten core targets. Among the top ten core proteins, AKT1, STAT3, MAPK14/P38, RELA/p65, and IL6 proteins exhibited much lower binding energies than other proteins, indicating that these five targets bound tightly to SJZT (Figure 3A, 3B). Therefore, given the molecular docking results and the roles of these targets in aging, we selected AKT1, STAT3, MAPK14/P38, RELA/p65, and IL6 proteins as the core targets.

### GO function and KEGG pathway enrichment analysis

**Figure 4:**
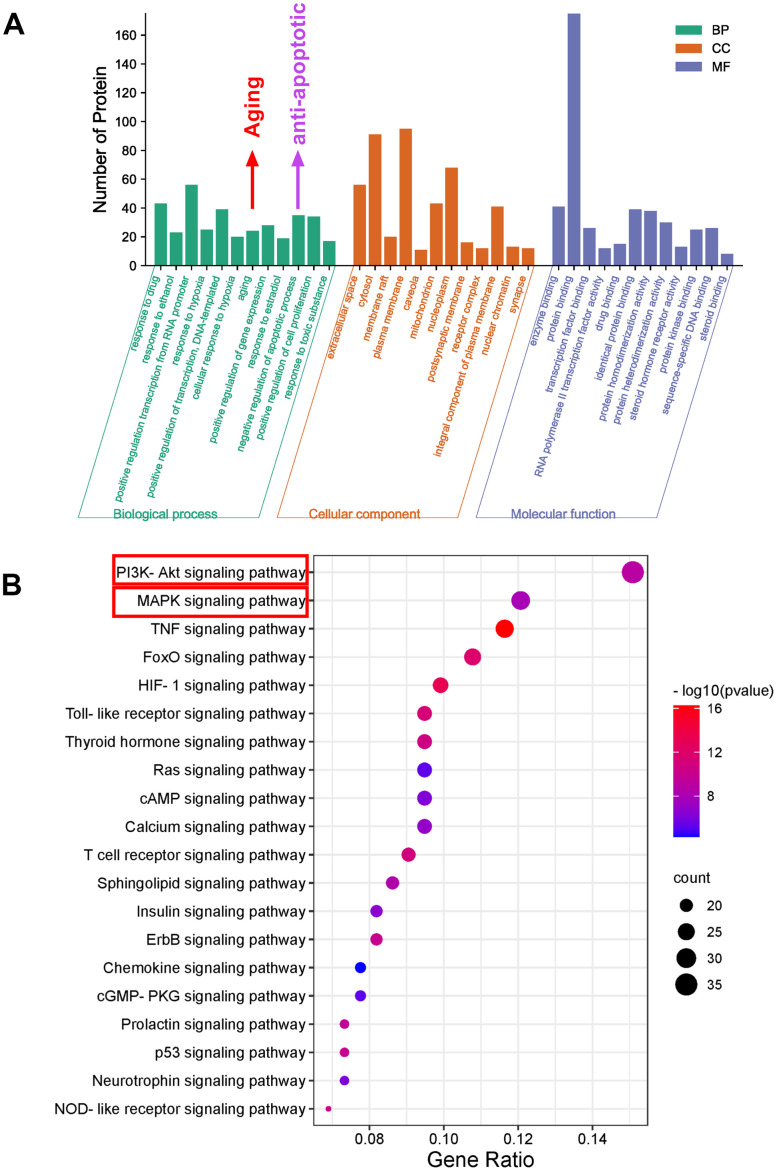
**Bioinformatics analysis of predicted targets.** (**A**) The distribution of GO entries in biological process, molecular function and cell composition (top 12 according to FDR < 0.05) are shown. (**B**) The top 20 KEGG pathways. The color scale indicates the different thresholds for the p-values, and the size of the dots represents the number of genes corresponding to each term.

To explore the potential anti-aging mechanism of SJZT, GO function and KEGG pathway enrichment analyses were performed. There were 454 GO entries according to an FDR<0.05, including 341 biological process terms, 42 cell composition terms and 71 molecular function terms (Supplementary Table 3). As depicted in Figure 4A, the GO enrichment results showed that 235 hub targets were enriched in aging and antiapoptotic biological processes. Notably, the aging processes were more significant.

Because signalling pathways can act systematically against aging, we explored pathways in aging. Subsequently, we performed enrichment analysis of KEGG pathways on the 235 hub targets (Supplementary Table 4). The top 20 signalling pathways by P value are presented (Figure 4B). The KEGG results indicated that the PI3K-AKT and MAPK signalling pathways were the most important. To further verify these two pathways, we carried out biological verification.

### SJZT can improve the aging-related phenotype of aging mice

**Figure 5:**
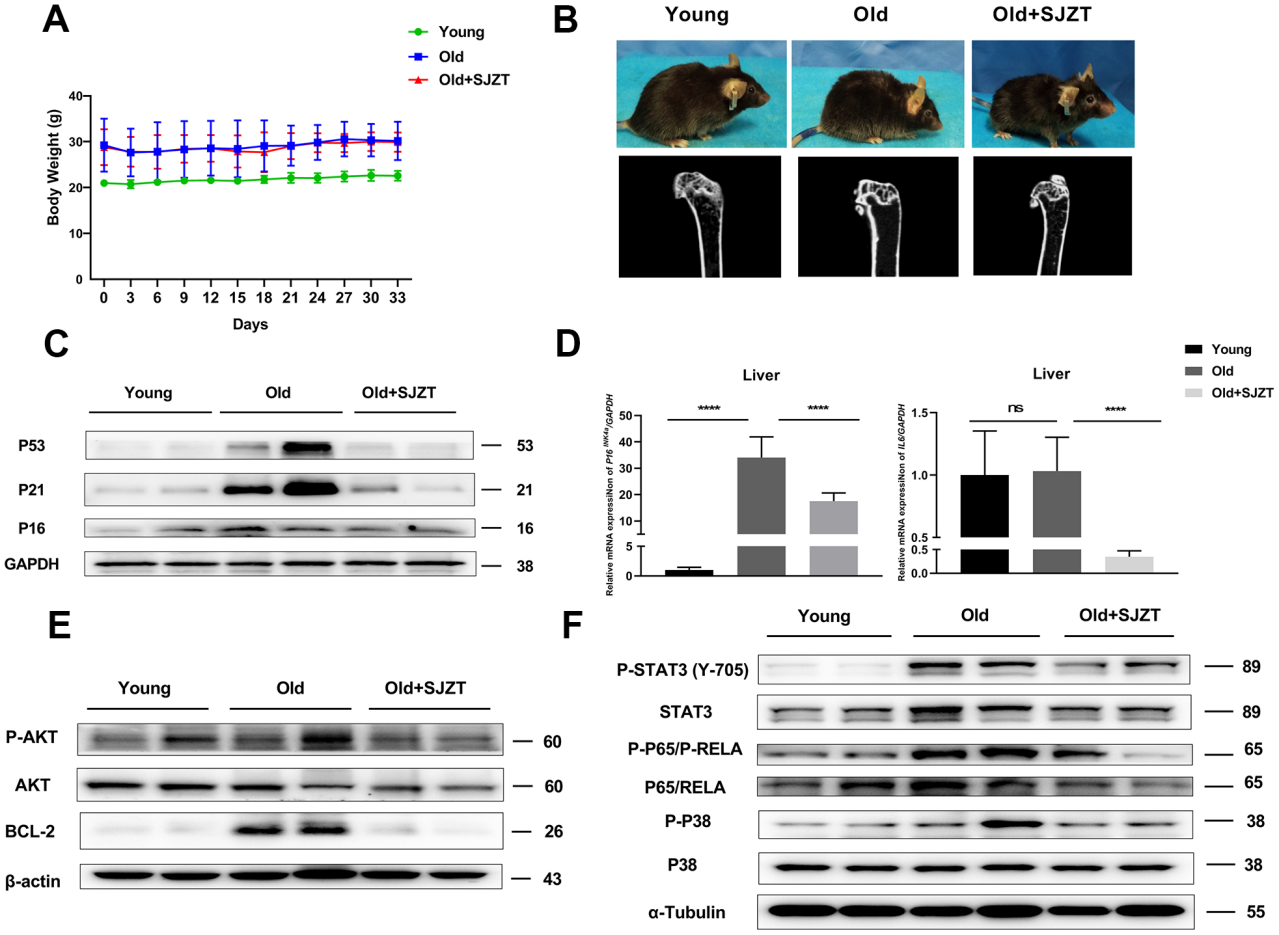
**SJZT can improve the aging-related phenotype of aging mice.** (**A**) Body weight of mice after SJZT or distilled water treatment. (**B**) Representative photos of the old mice and after SJZT treatment with considerable differences in skin and in hair. And μCT of the isolated shin bones to assess bone porosity. (**C**) The protein levels of P16, P21 and P53 determined by Western blots in mice liver tissue. (**D**) The mRNA levels of *p16* and *IL6* determined by qPCR in mice Liver tissue. (**E**, **F**) The protein expression levels of liver tissues were detected by western blot analysis. Data represent the means ± standard deviation. *P<0.05, **P<0.01 or ***P<0.001 (n=3).

To verify the results of network pharmacology, we used naturally aging mice to investigate the anti-aging effect of SJZT. Body weight measurements indicated that SJZT had no obvious toxicity (Figure 5A). Then, we assessed the aging-related phenotype after SJZT treatment. Hair loss is a typical symptom of aging in human skin [38, 39]. Rickets and hair shedding were effectively reduced in the SJZT group compared with the aging group. In addition, the micro-CT analysis results showed that the osteoporosis, which is a typical aging phenotype, was also improved after SJZT treatment. The total average bone mineral density was 431.6 mg/cm^3^ in the SJZT group and 394.5 mg/cm^3^ in the old group (Figure 5B). In addition, the protein expression of the senescence biomarkers P53, P21 and P16 was evaluated. As shown in Figure 5C, SJZT treatment significantly inhibited the expression of P53, P21 and P16. Similarly, we detected that the levels of aging-related mRNAs such as *P16* and *IL6*, were also significantly reduced in the SJZT treatment group (Figure 5D). Taken together, these data indicated that SJZT could improve the aging-related phenotype of aging mice by inhibiting the expression of P53, P21, *P16* and *IL6*.

### Effects of SJZT on the PI3K/AKT and P38 MAPK pathways in aging Mice

Considering the enriched biological process of SJZT in aging and anti-apoptosis (Figure 4A), a large number of studies have found that senescent cells have antiapoptotic properties [40, 41]. So we detected the expression of antiapoptotic proteins BCL2 and AKT. BCL2 and P-AKT levels were significantly reduced in the SJZT treatment group compared with the aging group (Figure 5E). The results indicated that SJZT had an obvious inhibit anti-apoptotic effect by inhibiting the protein expression of AKT and BCL2. Furthermore, the results of KEGG analysis also revealed enrichment in the PI3K-AKT signalling pathway.

To further validate the KEGG prediction of network pharmacology, we subsequently examined P38 MAPK signalling pathway members, including P38 (P-P38), STAT3 (P-STAT3) and p65 (P-p65). Senescent cells can release a host of proinflammatory mediators via the P38 MAPK signalling pathway [42]. As shown in Figure 5F, the phosphorylation levels of STAT3, P38 MAPK, and p65 were significantly reduced in the SJZT treatment group compared with the aging group. These results indicated that SJZT negatively regulated the activation of the P38 MAPK and PI3K-AKT signalling pathways to prevent aging.

## DISCUSSION

Many studies have shown that SJZT is a promising formula for treatment of aging related diseases [43–45]. However, the pharmacological mechanism of action of SJZT against aging is not yet fully understood. Thus far, many SJZT network pharmacology studies have been conducted only at the prediction level, and have lacked molecular docking and experimental verification [8, 46]. Hence, in this study, we performed a comprehensive analysis of network pharmacology coupled with *in vivo* experiments to further identify the underlying mechanisms and therapeutic targets of SJZT in aging effect.

Through a network pharmacology approach, we identified 131 potential active components and 235 hub targets. A compound-target network of SJZT demonstrated that SJZT compounds affected multiple targets. Moreover, SJZT compounds might possess overlapping targets, resulting in synergistic effects. GO enrichment analysis showed that 235 hub targets were enriched in aging and antiapoptotic biological processes. Our data indicated that the significance of the aging terms was extremely high, supporting our previous hypothesis. KEGG pathway enrichment analysis showed that SJZT combated aging through the inhibition of the PI3K-AKT signalling pathway and MAPK signalling pathway. Similar to previous studies [8], our study involved network pharmacological analysis, and PI3K-AKT signalling pathways were identified in the KEGG enrichment analysis. However, Liang et al. [8] did not consider Caco-2 entry in compound screening, and did not carry out molecular docking and biological experimental verification. To identify the active compounds needed for molecular docking, we performed further analysis and found that kaempferol and quercetin play important roles in the effects of SJZT. We found that kaempferol and quercetin bind most closely to the AKT1, STAT3, MAPK14/P38, RELA/p65, and IL6 proteins. The main active compounds (quercetin and kaempferol) have been reported to have direct anti-aging effects. For example, quercetin can downregulate p53, P21 and IL6, which are aging-related markers, to attenuate D-galactose induced aging in rats [47]. In addition, kaempferol decreases the expression levels of STAT3, CDK1, and PI3K/AKT/mTOR and P38MAPK signalling pathway members to inhibit the expression of the SASP [48]. In terms of secondary structure, quercetin has only one more hydroxyl group than kaempferol. They are similar in structure. This explains that the binding pockets of quercetin and kaempferol are roughly the same on the same protein, but the binding free energy and binding site are not necessarily the same (Supplementary Tables 5, 6). These results show that the strategy of applying network pharmacology to find potential active compounds is reliable and feasible.

Then, we designed *in vivo* experiments to confirm the hypothesis. First, there were no significant changes in the body weights of mice after administration of SJZT. However, treatment with SJZT could effectively reversed hair shedding and improved osteoporosis in mice. Second, to further verify whether SJZT had an anti-aging effect, we detected the expression of the P53, P21 and P16 proteins and the levels of *p16* and *IL6* mRNA in liver tissues. The levels of the above aging-related markers decreased significantly in mouse livers after administration of SJZT. The above *in vivo* experiment combined with molecular docking confirmed that IL6 and P53 were the core targets of SJZT in anti-aging in the PPI network. In addition, these results were consistent with the aging biological process of SJZT enrichment in GO analysis. Finally, we investigated whether the anti-aging role of SJZT involved the PI3K-AKT and P38 MAPK signalling pathways in the liver. In this study, the phosphorylation of the AKT, P38 MAPK, RELA/p65 and STAT3 proteins in liver tissues of the SJZT group was significantly decreased, indicating that the PI3K-AKT and P38 MAPK signalling pathways were inhibited after administration of SJZT. Similarly, these results combined with molecular docking validated AKT1, P38 MAPK, RELA/p65, P53, IL6 and STAT3 as were the core targets of SJZT in anti-aging.

**Figure 6:**
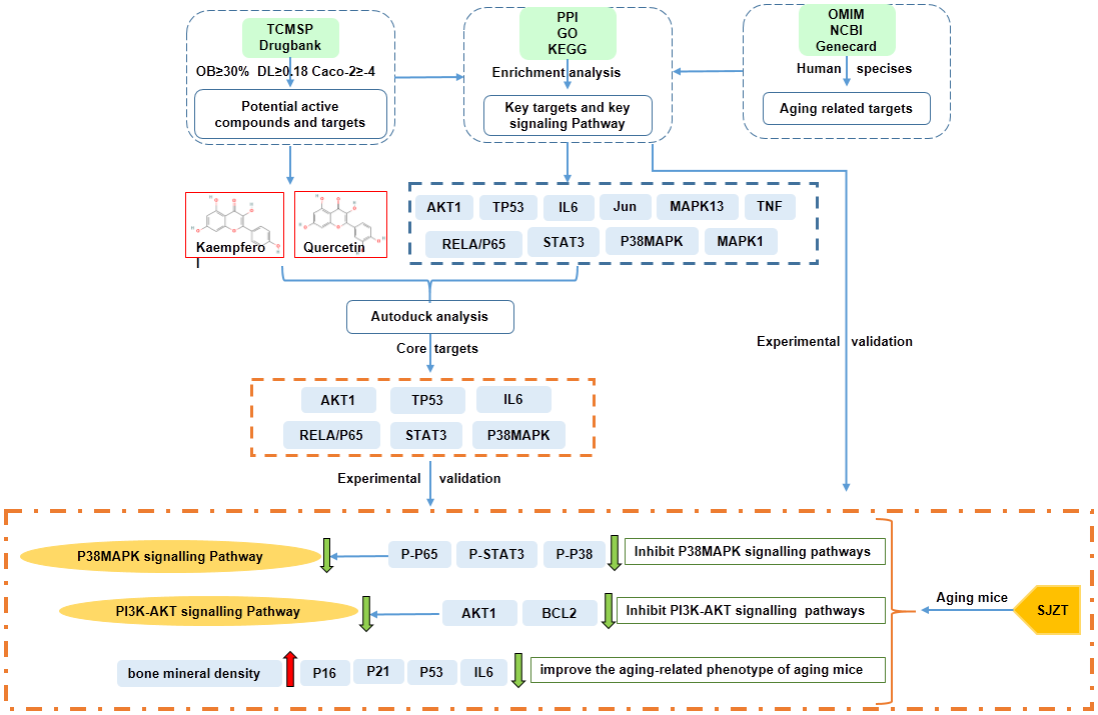
The mechanisms of SJZT in the treatment of aging by the network pharmacology approach and experimental validation.

In conclusion, the network pharmacological analysis of SJZT identified 131 bioactive compounds and 235 target genes associated with aging. AKT1, STAT3, TP53, RELA/p65, P38MAPK and IL6 were revealed as the core targets of SJZT in anti-aging. According to the results of GO and KEGG enrichment analyses, we verified that SJZT exerts an anti-aging effect in mice by downregulating the P38 MAPK and PI3K-AKT signalling pathways. At the same time, SJZT can improve the aging-related phenotype by inhibiting aging-related markers (Figure 6). However, other signalling pathways (e.g., the Toll-like receptor signalling pathway, and HIF-1 signalling pathway) may also be involved in the anti-aging effect of SJZT. In a follow-up experiment, we will do metabonomic analysis, such as HPLC to determine the active components and potential targets of SJZT.

## Supplementary Material

Supplementary Table 1

Supplementary Table 2

Supplementary Table 3

Supplementary Table 4

Supplementary Tables 5 and 6
