# Supplementary material for: Elucidating the anti-aging mechanism of Si Jun Zi Tang by integrating network pharmacology and experimental validation in vivo
Source: Aging (Albany NY). 2022 May 10;14(9):3941–55. doi: 10.18632/aging.204055 (PMC9134961; doi:10.18632/aging.204055)
Supplement: Supplementary Tables 5 and 6 [file aging-14-204055-s005.pdf]

SUPPLEMENTARY TABLES

Supplementary Table 5. Molecular docking binding energy score.

Protein name	PDB ID	Quercetin binding energy score (kcal/mol)	Kaempferol binding energy score (kcal/mol)
AKT1	4gv1	-8.3	-8.1
STAT3	6njs	-7.5	-7.1
JUN	1a02	-5.6	-5.6
MAPK3	4qtb	-9.3	-9.1
TP53	4iby	-6.8	-6.3
MAPK1	6slg	-8.1	-6.1
TNF	7jra	-6.8	-6.8
RELA	6nv2	-7.2	-6.9
MAPK14	3zs5	-11	-10.7
IL6	1alu	-7.1	-6.7

Supplementary Table 6. Relevant coordinate of the docking box.

Compounds-Protein	Grid size X, Y, Z	X, Y, Z coordinates
Quertein-AKT1	32, 44, 52	-23.423, 5.856, 11.606
Quertein-STAT3	52, 90, 54	9.299, 54.795, 2.625
Quertein-MAPK14	42, 46, 56	22.544, 31.878, 17.449
Quertein-RELA/P65	40, 42, 64	27.293, 13.587, 2.039
Quertein-IL6	104, 118, 84	2.669, -19.932, 3.112
Kaempferol-AKT1	32, 44, 52	-23.423, 5.856, 11.606
Kaempferol-STAT3	52, 90, 54	9.299, 54.795, 2.625
Kaempferol-MAPK14	42, 46, 56	22.544, 31.878, 17.449
Kaempferol-RELA/P65	40, 42, 64	27.293, 13.587, 2.039
Kaempferol-IL6	104, 118, 84	2.669, -19.932, 3.112
